# Evolutionary Origin of the P2X7 C-ter Region: Capture of an Ancient Ballast Domain by a P2X4-Like Gene in Ancient Jawed Vertebrates

**DOI:** 10.3389/fimmu.2020.00113

**Published:** 2020-02-06

**Authors:** Airi Rump, Olli Pekka Smolander, Sirje Rüütel Boudinot, Jean M. Kanellopoulos, Pierre Boudinot

**Affiliations:** ^1^Immunology Unit, Department of Chemistry and Biotechnology, Tallinn University of Technology, Tallinn, Estonia; ^2^Department of Chemistry and Biotechnology, Tallinn University of Technology, Tallinn, Estonia; ^3^Institute for Integrative Biology of the Cell (I2BC), CEA, CNRS, Université Paris-Saclay, Gif-sur-Yvette, France; ^4^Virologie et Immunologie Moléculaires, INRAE, Université Paris Saclay, Jouy en Josas, France

**Keywords:** purinercic receptors, P2X7, evolution, ballast domain, NANOR-like proteins

## Abstract

P2X purinergic receptors are extracellular ATP-gated ion channel receptors present on the cell plasma membrane. P2X receptors have been found in Metazoa, fungi, amoebas, and in plants. In mammals, P2X7 is expressed by a large number of cell types and is involved in inflammation and immunity. Remarkably, P2X7 does not desensitize as other P2X do, a feature linked to a “C-cysteine anchor” intra-cytoplasmic motif encoded by exon 11. Another specific feature of P2X7 is its C-terminal cytoplasmic ballast domain (exon 13) which contains a zinc (Zn) coordinating cysteine motif and a GDP-binding region. To determine the origin of P2X7, we analyzed and compared sequences and protein motifs of the C-terminal intra-cytoplasmic region across all main groups of Metazoa. We identified proteins with typical ballast domains, sharing a remarkably conserved Zn-coordinating cysteine motif. Apart from vertebrates, these ballast domains were not associated with a typical P2X architecture. These results strongly suggest that P2X7 resulted from the fusion of a P2X gene, highly similar to P2X4, with an exon encoding a ballast domain. Our work brings new evidence on the origin of the P2X7 purinergic receptor and identifies the Zn-coordinating cysteine domain as the fundamental feature of the ancient ballast fold.

## Introduction

The P2X7 receptor is the seventh member of the P2X receptor family of ATP-gated cation channels. Brief activation of P2X7R with extracellular ATP in its tetra-anionic form, ATP^4−^, opens cation-specific ion channels. P2X7 activation requires higher ATP concentrations (0.5 to 1 mM) than for other members of the P2XR family; required concentrations are nanomolar for P2X1 and P2X3, and micromolar for P2X2 and P2X4. In addition, P2X7R does not desensitize while the other P2X receptors like P2X1 and P2X3 desensitize rapidly or like P2X4 for which the desensitization is not as fast. Furthermore, prolonged ligation of P2X7 results in the formation of non-selective pores in the plasma membrane, permeable to molecules up to 900 Da.

Prolonged ATP ligation of P2X7 can trigger membrane blebbing (1) and cell death by apoptosis (2) or lysis/necrosis (3–6) depending on the cell type. In several cell types, for example in macrophages activated by bacterial products, the main cell death induced via P2X7R stimulation is pyroptosis [(7–9), reviewed in (10)]. However, several studies have shown that P2X7 is able to stimulate growth or promote survival (11, 12). Besides its involvement in cell death or proliferation, P2X7 triggers several biochemical pathways, leading to rapid release of mature IL-1β and IL-18 from macrophages (13, 14), killing of various intracellular pathogens in macrophages (15, 16), and proteolytic cleavage of plasma membrane proteins such as L-Selectin, CD23, TNFα, CD27, matrix metalloproteinase-9, interleukin-6 receptor, and the amyloid precursor protein (17–22). The role of P2X7 in inflammation and infectious diseases has been the subject of numerous studies and has been reviewed thoroughly (10, 23).

One striking feature of P2X7 is its ability to open a non-selective ≪ macropore ≫ after repetitive or prolonged stimulation by ATP. The nature of this non-selective pore has been the subject of numerous studies and remains controversial [reviewed in (24)]. It was hypothesized that P2X7 is able to dilate and form the pore or that non-selective pore formation requires additional molecules such as connexin 43 (25), pannexin-1 (26), or anoctamin 6 (27). However, Karasawa et al. have recently incorporated highly purified panda P2X7 into liposomes and found that ATP stimulation triggered the non-selective pore formation in the absence of other proteins. In addition, a cysteine rich motif, containing C362 and C363, is required for the non-selective macropore opening. These cysteines are palmitoylated, and their mutation to serine abolishes P2X7 capacity to form the macropore (28). The main conclusion of this work is consistent with studies showing that P2X7 stimulation triggers the formation of a non-selective macropore in macrophages from pannexin-1 or connexin 43 knock-out mice (29, 30).

The first crystallographic structure of a P2X receptor showed that zebrafish P2X4 is organized as a homotrimer of P2X4 subunits (31). Crystal structures of a truncated panda P2X7 in the presence of five different antagonists were later reported. They bind to the same hydrophobic pocket away from the ATP binding site acting as allosteric non-competitive inhibitors (32). A major breakthrough was recently achieved by McCarthy et al. (33) who published the first complete structure of the rat P2X7 receptor obtained by single-particle cryoelectron microscopy. The structure of the carboxy-terminal portion of the P2X7 receptor, which is unique to this P2X, defines a novel fold called ≪ ballast ≫ which contains a dinuclear Zn ion complex and a pocket containing a guanosine nucleotide.

P2X genes have been identified across eukaryotes, for example in Metazoa, fungi, amoebas, and plants (34). These receptors share well-conserved structural elements and are activated by ATP (35). Among Metazoa, most species have one or two P2X genes. However, they have been apparently lost in some groups such as insects and nematodes. Jawed vertebrates generally possess seven conserved types of P2X, including P2X7. P2X7 and P2X4 genes are closely linked and encode highly similar membrane receptors (36). While the extracellular domains of all P2X receptors are highly similar, it is important to note that the long intracytoplasmic region of P2X7 was not found in any other P2X, either in vertebrates or other species. Little is known about the function of P2X7 in non-mammalian species. However, P2X7 identified in Ayu (*Plecoglossus altivelis*, an Asian salmoniform), is induced by infection and is involved in ATP dependent cell death, phagocytosis, and bactericidal activity of macrophages (37, 38). Importantly, after transfection in HEK293 cells, seabream or zebrafish P2X7 receptors were unable to induce the maturation and secretion of human or fish IL-1β. However, the chimeric P2X7 receptor composed of the extracellular domain of the seabream P2X7 linked to the intracellular region of the rat P2X7 triggers the maturation and release of both types of IL-1β. These experiments pointed to functional differences between intracellular parts of the rat and fish receptors (39).

In this work, we focused on the carboxy-terminal, intracytoplasmic sequence of P2X7 receptor and its conserved motifs including Zn-coordinating set of Cysteines and the GDP binding domain identified in McCarthy et al. (33). We looked for proteins comprising related domains within vertebrates and beyond. We identified the primordial module from which the P2X7 carboxy-terminal region originated and found its representatives across the main groups of Metazoa.

## Materials and Methods

### Identification and Analysis of Counterparts of the Intracytoplasmic Domain of P2X7

Available EST indices and genome databases were mined using TBLASTN and human or rat P2X7 intracytoplasmic sequences as queries. Searches in EST databases were mainly performed at http://www.ncbi.nlm.nih.gov/. Blast queries on complete genomes were sent to http://www.ensembl.org, http://www.ncbi.nlm.nih.gov/ and http://reefgenomics.org/blast. When relevant genomic regions were identified, potential exons were identified by comparison with known sequences, consensus nucleotide sequences were translated and ORF were compared to predicted protein models. Multiple alignments were performed using Clustal Omega (https://www.ebi.ac.uk/Tools/msa/clustalo/), to analyse the conservation of key residues identified previously in the P2X7 ballast domain. Putative domains and motifs were analyzed based on literature and sequence analysis using Interproscan and Smart programs. Phylogenetic analyses were performed using MEGA version 7 (40).

### Linkage Analysis

The next five markers were studied upstream and downstream of each gene containing domains homologous to P2X7 ballast. Sets of paralogs and syntenic homologs of these markers were identified combining the phylogenetic relationships available at Ensembl Metazoa (http://metazoa.ensembl.org/index.html), Genomicus Metazoa (http://www.genomicus.biologie.ens.fr/genomicus-metazoa-30.01/cgi-bin/search.pl) and direct tblastn queries on relevant genomes. Their location relative to homologous ZCD-containing genes within the same species or across species was analyzed to look for conserved synteny set.

### Molecular Modeling of the Structure of P2X7 Homologs

Protein structure homology-modeling was performed using the SWISS-MODEL program, accessible via the ExPASy web server (https://swissmodel.expasy.org/), using as template the structure of the rat P2X7 receptor obtained by single-particle cryoelectron microscopy [PDB ID: 6u9v (33)]. The relevant domains of the models were extracted and compared using Pymol (available at https://pymol.org/2/).

## Results

### P2X7 C-Terminal Region Comprises a Conserved Zn-Coordinating Cysteine Based Domain

**Figure 1:**
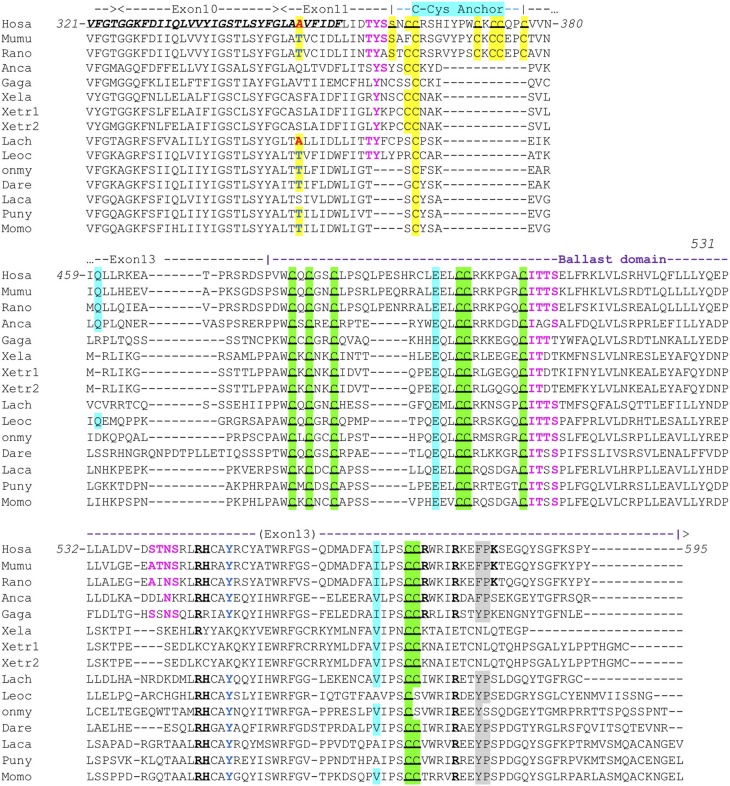
Conserved motifs in P2X7 C-terminus from representative vertebrate species. Exon limits and functional domains are indicated above sequence alignment. Positions within the human P2X7 protein are indicated. The second TM domain is in bold italic and underlined. Key residues of the C-Cyst Anchor are boxed and represented in black on yellow background (33). Zn-coordinating cysteines which define ZCD are boxed and highlighted in green (33). Key residues of the GDP binding motif are in bold underlined (33). Conserved Y_550_ is in blue (41, 42), F/Y_581_P_582_ are highlighted on gray background (43), β arrestin binding sites [T_357_YSS, I_507_TTS, A_540_TNS (42)] are in purple and bold. Residues Q460, E496, and I568 are highlighted on light blue background. Their importance is based on the identification of three loss of function polymorphisms of the human P2X7 gene, Q460R, E496A, and I568N (44). Interestingly, the A348 residue (in red on yellow background) is replaced by a T in several mammal and fish species (in blue on yellow background), indicating it constitutes an ancestral variation. In human, the A348T substitution is associated to a major gain of function (45). Sequences: Mammals: Human, *Homo sapiens* (hosa, ENSG00000089041); Mouse, *Mus musculus* [mumu (ENSMUSG00000029468)]; Rat, *Rattus norvegicus* (rano, ENSRNOG00000001296). Reptiles and birds: Carolina anole, *Anolis carolinensis* (anca, ENSACAG00000022072); Chicken, *Gallus gallus* (gaga, ENSGALG00000003863). Amphibians: Clawed frog, *Xenopus laevis* (xela, NP_001082196); *Xenopus tropicalis* (xetr1: ENSXETG00000001030, xetr2: ENSXETG00000001030) Crossopterygians: Coelacanth, *Latimeria chalumnae* (lach, XP_005989088). Bony fishes: Spotted gar, *Lepisosteus ocelatus* (leoc, ENSLOCG00000006925); Rainbow trout *Oncorhynchus mykiss* (Onmy; XP_021461359); Zebrafish, *Danio rerio* (dare, ENSDARG00000042440); Barramundi perch, *Lates calcarifer* (laca, ENSLCAG00010007049); Makobe Island cichlid, *Pundamilia nyererei* (puny, ENSPNYG00000007433); Sunfish, *Mola mola* (Momo, ENSMMOG00000014797).

**Figure 2:**
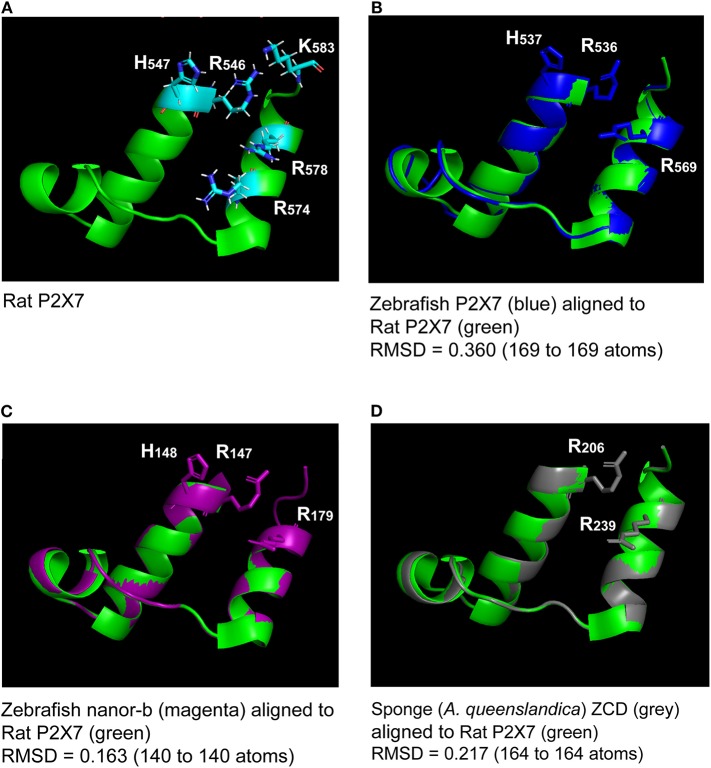
Superimposition of the rat P2X7 GDP binding domain **(A)** with molecular models of the homologous region from zebrafish P2X7, zebrafish nanor-b, and sponge ZCD-containing protein **(B–D)**. Lateral chains of the rat GDP coordinating amino-acids (R546, H547R574, R578, and K583) are shown **(A)**. Lateral chains are also represented for conserved residues in the other sequences **(B–D)**. The green color refers to the template, i.e., to the rat structure. Models of zebrafish P2X7 **(B)**, zebrafish nanor-b **(C)** or sponge ZCD containing protein **(D)** GDP binding regions (in blue, magenta, and gray, respectively) were superimposed to the structure of rat P2X7 (in green). The least Root Mean Square Deviation (lRMSD) is indicated with the number of atoms used for its computation. The rigid motion used to compute the lRMSD was used to superimpose the structures. Computed by the iterative aligner from Pymol.

All human P2X sequences contain a typical P2X motif (Interpro IPR001429) encoded by exons 1–10 (Figures S1A,B). In this region, exon junctions are highly conserved between the different P2X. In contrast, sequences of P2X C-terminal regions are very variable, with various number of exons and different positions of exon junctions. Thus, exon 12 is not related between P2X, and only P2X5 and P2X7 have a 13th exon: a short one for P2X5 encoding 13 aa, and a long one for P2X7 encoding a 170 aa peptide with multiple conserved cysteines which were shown to be in a tetrahedral geometry likely coordinating a dinuclear Zn ion complex [Figure S1B (33)]. We then compared P2X7 sequences across vertebrates, from fin fish to mammals. While we could not find any typical P2X7 in agnathans or in chondrichthyans (sharks and rays), bony fish species have typical P2X7, as well as all studied tetrapods. The P2X motif was highly conserved in these sequences (Figure S1A). In contrast, the 3′ end of exon11 and the 5′ end of exon 13 are not well-conserved across vertebrate P2X7. Interestingly, the palmitoylation site [the “C-cys anchor” from McCarthy et al. (33)] located in exon11 in rat P2X7 (motif **S**N**CC**RSHIYPW**C**K**CC**QP**C**) is not conserved across vertebrates (Figure 1). This motif is present across mammals (both in eutherians and marsupials, Figure S2), although not fully conserved in the elephant. The most conserved part is the initial SxxCC motif, which is also present in some reptiles/birds as well as in Xenopus, while the end of the motif is lost in these species Figure 1. In teleost fish, a unique cysteine is conserved at the position of the human C-Cys anchor, but in a different context (LIGTGCYSK). In the spotted gar, which belongs to a basal branch of the fish lineage, the motif is different (FITTYLYPRCCAR), suggesting that the one found in teleosts may have evolved secondarily. In brief, only one C of the exon 11 motif can be found from mammals to fish, and might constitute a conserved palmitoylated site. Exon12 is relatively well-conserved, but does not contain cysteine. In exon 13, encoding the so-called “ballast domain” (33), Zn-coordinating cysteines are overall extremely well-conserved in P2X7 from fish to mammals (Figure 1). However, the 5′ side of the exon is highly variable, in length as well as in sequence. This region does not contain any position with a residue present in all species analyzed in Figure 1 and Figure S1A. It is particularly long in cyprinids as observed both in zebrafish (dare) and goldfish (not shown), in EST as well as in genomic sequences. The region encoded by the 3′ end of exon 13 of rat P2X7 also contains a high-affinity guanosine nucleotide binding site (R_546_-H_547_,R_574_xxR_578_xxxxxK_583_) (33). Interestingly, the R_546_-H_547_ motif is well-conserved across vertebrates except frogs (Figure 1), and R_578_ is present in most analyzed species. We then used SwissModel to produce structural models of this region from zebrafish P2X7, using the cryo-EM structure of the rat P2X7 (33) as a template. Superimposition of this model with the structure of the human GDP-binding region shows a very good fit (Figures 2A,B), with the conserved residues standing in similar positions. It is important to note that the region between the R_546_-H_547_ and the R_574_R_578_K_583_ motifs is overall well-conserved, with a WRF motif always present across vertebrate P2X7 (Figure 1). Thus, although residues coordinating GDP in rat are not all conserved in fish, the structure of this part of the protein may remain compatible with GDP binding.

Based on these data, we therefore defined a highly conserved region located in exon13 which contains most of the Zn-coordinating cysteines in human P2X7. The three clusters of cysteines have the following consensus sequences: (1) the first one contains the motif PxW**C**x**C**x_2_**C**, (2) the second one L**CC**Rx3Gx**C**ITTS/T (3) the last motif is composed of (L/I/V)PS**C**(**C**/S)x_3_IRx_2_(F/Y)Px_5_Y(S/T)G. This region—we name “Zn-coordinating cysteine based domain” (ZCD) hereafter—contains seven/eight conserved C residues (Figure 1) but does not comprise the C-cys anchor motif.

### P2X7 ZCD Is Found in Only a Few Other Proteins Within Vertebrates

**Figure 3:**
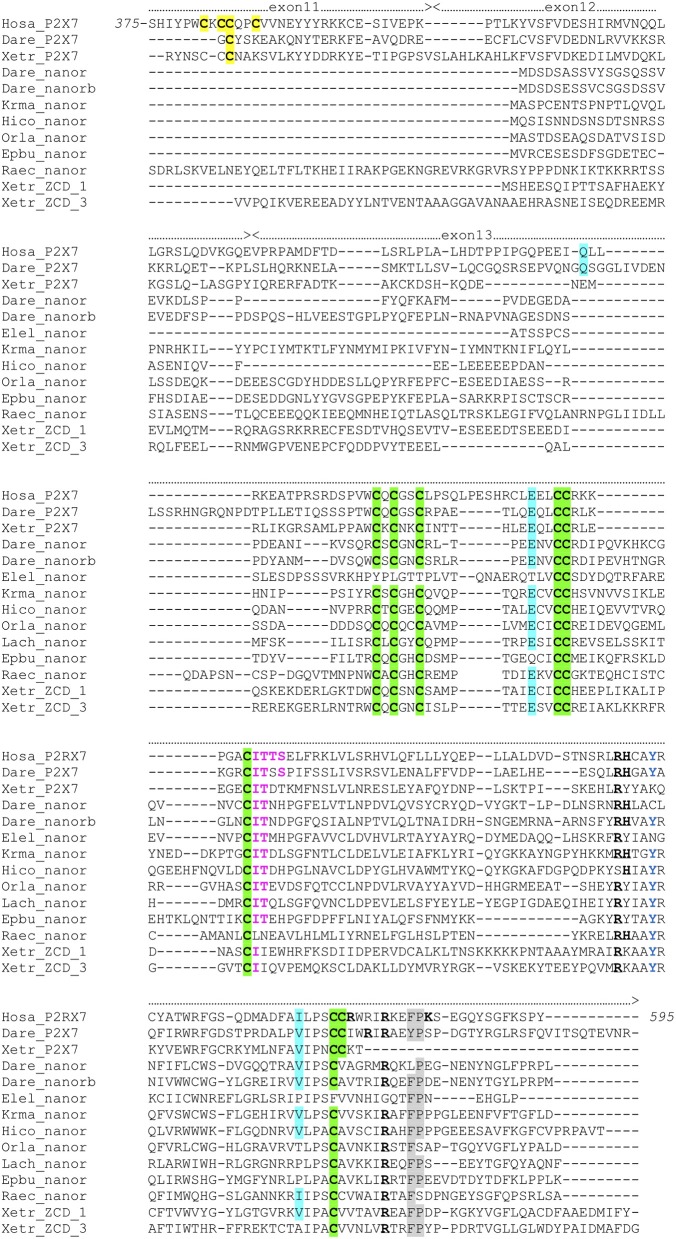
Multiple alignment of P2X7 Cter region with nanor proteins, and frog ZCD-containing proteins. Exon limits and functional domains are indicated above sequence alignment. Positions within the human P2X7 protein are indicated. Key residues of the C Cyst Anchor are boxed and represented in black on yellow background in P2X7 sequences. Zn-coordinating cysteines which define ZCD are boxed and highlighted in green. Key residues of the GDP binding motif are in bold underlined. Conserved Y_550_ is in blue, F/Y_581_P_582_ are highlighted on gray background, the β arrestin binding site I_507_TTS, is in purple and bold. Residues Q460, E496 and I568 are highlighted on blue background. Their importance stems from the identification of three loss of function polymorphisms of the human P2X7 gene: Q460R, E496A, and I568N (44). Sequences: Hosa_P2X7, human (ENSG00000089041); Leoc_P2X7, spotted gar (ENSLOCG00000006925); Dare_P2X7, zebrafish (ENSDARG00000042440); Xetr_P2X7, Tropical clawed frog (ENSXETG00000032525), Dare_nanor, zebrafish (ENSDARG00000058917); Dare_nanor-b, zebrafish (ENSDARG00000076264); Elel_nanor, Electric eel (ENSEEEG00000011457); Krma_nanor, mangrove rivulus (ENSKMAG00000012512); Hico_nanor, tiger tail seahorse (ENSHCOG00000018472); Orla_nanor, japanese medaka (ENSEBUG00000006500); Lach_nanor, coelacanth (ENSLACG00000022457); Epbu_nanor-like, Hagfish (ENSEBUG00000006500); Raec_nanor, little skate (GH269666); Xetr_Palm_ 1, Tropical clawed frog, A0A1B8XY14; Xetr_Palm_3, Xenopus, EL845305.

To get more functional insights about P2X7 C-terminal region, we first looked for its association with other domains across vertebrate proteomes. To this purpose, we first performed blast searches using the human P2X7 ZCD as bait. We mainly detected P2X7 proteins in all tested tetrapods and in most bony fish genomes. However, in zebrafish, two additional proteins were detected, which contains only ZCD: nnr (nanor; ENSDARG00000058917; chromosome 15:1589899) and nanor-b (ENSDARG00000076264; chromosome 22:4797625). These proteins also showed a very well-conserved ZCD motif, as found in P2X7 (Figure 3). Nanor genes have Ensembl orthologs in several other fish species, in the coelacanth and in an agnathan, the hagfish. It seems that nanor-like genes are duplicated in most species in which they are present (Figure S3). Considering their genomic context, only two markers were conserved close to nanor-like paralogs: one between medaka and mangrove rivulus, and one between electric eel and medaka. Sequences with a significant level of similarity were found close to nanor genes in other fish species, but they were not true orthologs (Figure S3). Thus, nanor and related genes apparently do not belong to a synteny block conserved across vertebrate groups, not even across bony fish. No conserved synteny was detected between nanor and p2X7 genes either. Further blast searches identified also additional sequences with ZCD in cartilaginous fish: in a ray (*Raja erinacea;* GH269666), and in the elephant shark (*Callorhinchus milii*; ENSCMIG0000001795).

**Figure 4:**
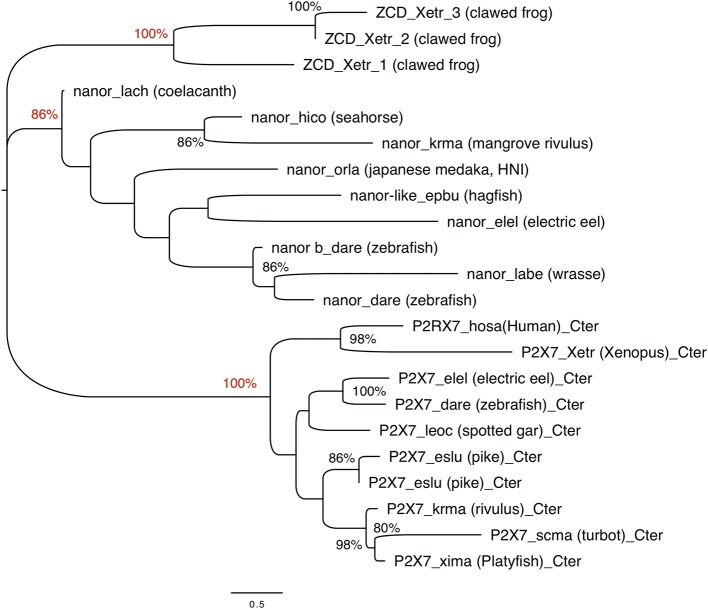
Phylogenetic analysis of P2X7 and nanor-like sequences from vertebrates. The optimal tree (Neighbor-Joining method, 100 bootstrap) is shown. Bootstrap values higher that 80% are indicated at nodes, and key positions are in red. The distances were computed by Mega6 using the JTT matrix-based method and are in the units of the number of aa substitutions per site. Sequences are as follows: nanor_dare (zebrafish, ENSDARG00000058917); nanor b_dare (zebrafish,ENSDARG00000076264); nanor_labe (ballan wrasse,ENSLBEG00000021957); nanor_elel (Electric eel, ENSEEEG00000011457); nanor-like_epbu (Hagfish, ENSEBUG00000006500);nanor_orla (japanese medaka, ENSEBUG00000006500); nanor_krma (mangrove rivulus, ENSKMAG00000012512); nanor_hico (tiger tail seahorse; ENSHCOG00000018472); nanor_lach (coelacanth, ENSLACG00000022457); ZCD-containing protein 1,ZCD_xetr_1, A0A1B8XY14; ZCD-containing protein 2, ZCD_xetr_2, F6R9E3; ZCD-containing protein 3, ZCD_xetr_3, EL845305; P2X7_hosa, human (ENSG00000089041); P2X7_xetr, xenopus tropicalis (ENSXETG00000032525), P2X7_leoc, spotted gar (ENSLOCG00000006925); P2X7_dare (zebrafish) (ENSDARG00000042440); P2X7_elel, Electric eel (ENSCMIG00000006667);P2X7_eslu, pike (ENSELUG00000001869); P2X7_eslu, pike (ENSELUG00000018695); P2X7_krma, mangrove rivulus(ENSKMAG00000003524); P2X7_scma turbot (ENSSMAG00000016255); P2X7_xima platyfish (ENSXMAG00000016836).

The ZCD present in all these proteins actually defines a Panther family (PTHR36981). While blast search did not find any obvious counterpart of nanor sequences in tetrapods, three sequences from Xenopus belong to the Panther family PTHR36981 (Figure 3). Phylogenetic analyses identified three clusters of ZCD-containing proteins: (1) typical P2X7 found from bony fish to mammals, (2) nanor-like proteins, which contain only the Zn-coordinating domain and a short additional N-ter region, and (3) three Xenopus proteins of similar structure, without P2X-like domain, which form a distinct group (Figure 4). Although these frog sequences do not contain an N-terminal P2X motif, they are not highly similar to fish nanor sequences, and are in fact more distant from them than the hagfish nanor-like sequence.

Key residues for GDP binding identified in rat P2X7 are not all conserved in nanor-like proteins. Residues homologous to rat R546H547 and R578 are quasi-conserved, H being sometimes replaced by Y (Figure 3). In contrast, rat R574 and K583 which are not present in amphibians and fish P2X7, are also absent in nanor sequences. However, superposition of the Swissmodel of the region from zebrafish nanor-b with its counterpart in rat P2X7 suggests that these structures may be rather similar, allowing GDP binding (Figure 2C).

Altogether, these data show that the ZCD seen in vertebrate P2X7 is also found in a few shorter proteins containing no other domains, in species belonging to Agnathans, Chondrichtyans, bony fish and tetrapods (in Amphibians). However, these genes are apparently absent in amniotes.

### The P2X7 ZCD Is an Ancient Module Present Across All Main Divisions of Metazoa

**Figure 5:**
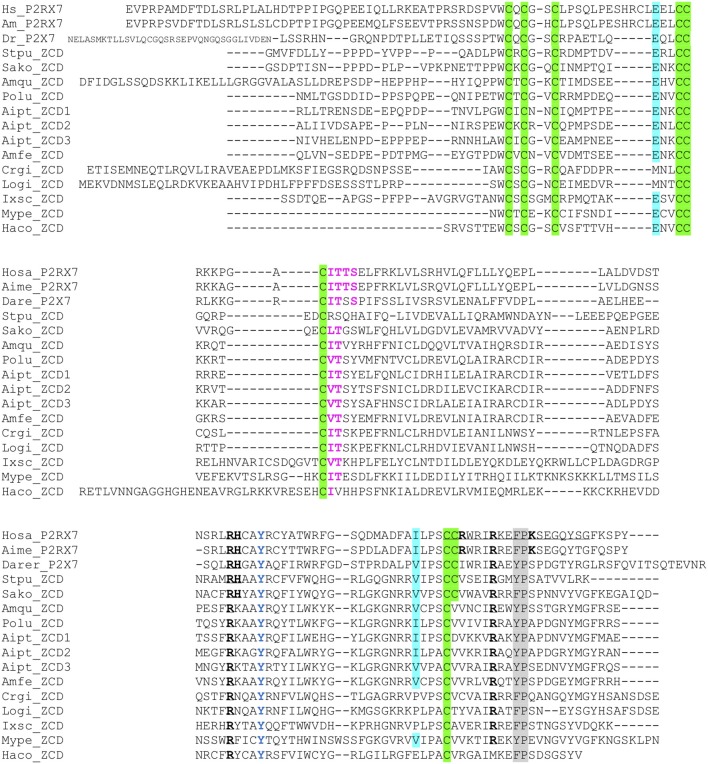
Multiple alignment of ZCD domains across Metazoa. Zn-coordinating cysteines which define ZCD are boxed and represented in black on green background. Key residues of the GDP binding motif are in bold underlined. Conserved Y_550_ is in blue, F/Y_581_P_582_ are highlighted on gray background, the β arrestin binding site I_507_TTS is in purple and bold. Residues E496 and I568 are highlighted on blue background. Their importance stems from the identification of two loss of function polymorphisms of the human P2X7 gene: E496A and I568N (44). Sequences: from Deuterostomes [Hosa_P2X7, human (ENSG00000089041); Rano_P2X7, rat (ENSRNOG00000001296); Aime_P2X7, panda (ENSAMEG00000014102); Dare_P2X7, zebrafish (ENSDARG00000042440); Stpu_ZCD, sea urchin (SPU_000568); Sako_ZCD, accorn worm (XP_002733146)]; from Protostomes [Crgi_ZCD, oyster (Cg_CGI_10024238); Logi_ZCD, Lottia (LotgiG163261); Ixsc_ZCD, mite (XP_029850345), Mype_ZCD, a hemiptere insect (XP_022182386); Haco_ZCD, a *Haemonchus* nematode (VDO68347)]; and from basal groups of Metazoa [Amqu_ZCD, sponge (Aq_Aqu2.1.01189); Polu_ZCD, *Porites lutea* (Pl_ut2.m8.33056, see http://reefgenomics.org/blast/#result) (Cnidaria); Aipt_ZCD, *Aiptasia* sp., AIPGENE13307 (Cnidaria) and Amfe_ZCD, *Amplexidiscus fenestrafer* (scaffold 1288, see http://reefgenomics.org/blast/#result) (Cnidaria)].

We then looked for ZCD in proteins from other groups of Metazoa. Within deuterostomians, such sequences were found in an echinoderm, the sea urchin *Strongylocentrotus purpuratus*, and in the acorn worm *Saccoglossus*. All Zn-coordinating cysteines found in P2X7 and nanor-like sequences were conserved in these proteins (Figure 5). In protostomes, no such domain could be detected from *C. elegans* or *Drosophila* databases. However, ZCD was found in proteins from the oyster *Crassostrea gigas* and from the limpet *Lottia gigantea*, indicating that it is present in mollusks. Among Ecdysozoa, the motif was found in arthropods–in the mite *Ixodes scapularis* and in a true bug (*Myzus persicae*)—as well as in nematods *Haemonchus contortus* and *Ancylostoma caninum* (Figure 5). Surprisingly, it may be absent from holometabole insects since we could not find it in dipterans (flyes), hymenopterans (ants, bees and wasps) or coleopterans (beetles). ZCD was also found in a sponge (*Amphimedon queenslandica*) and in a number of Cnidaria (Figure 5), but not in *Trichoplax adhaerens* (a placozoan) or in *Mnemiopsis leidyi* (a ctenophore). All these ZCD containing proteins belong to the Panther family PTHR36981, as nanor and nanor-like proteins. The Zn-coordinating cysteines were remarkably conserved in all these sequences. In contrast, among residues coordinating the binding of GDP in rat, only R546 and R578 were conserved. As for zebrafish P2X7 and NANOR-B, we superimposed the structure of this region from rat P2X7 to a model build from the sponge ZCD. Figure 2D shows that the conserved R residues stand in similar configuration in the model and in the rat structure. Although H547, R554, and K583 were not found in invertebrates, the region comprises several highly conserved positions including: A549, Y550, Y/F553, W559 I/V568, P570, and C572 (Figure 5). Our data therefore suggest that the structure of this region in the sponge ZCD may be conserved and may also bind a GDP.

As noted above for vertebrates, only one or a few (<10) ZCD-containing genes were found in each species. ZCD was typically found in relatively short proteins without other conserved domains and without transmembrane region, in contrast to P2X7. The only exceptions were found in the mite *Ixodes scapularis* in which two ORF comprised, respectively, a TolA (XP_029850345) or TAHP(XP_029850348) domain at the N-terminus, with the Zn-coordinating cysteines region at the C-terminus. The ZCD domain is always found at the C-terminus of the protein, while the N-terminal parts were of variable length and without obvious homologs.

**Figure 6:**
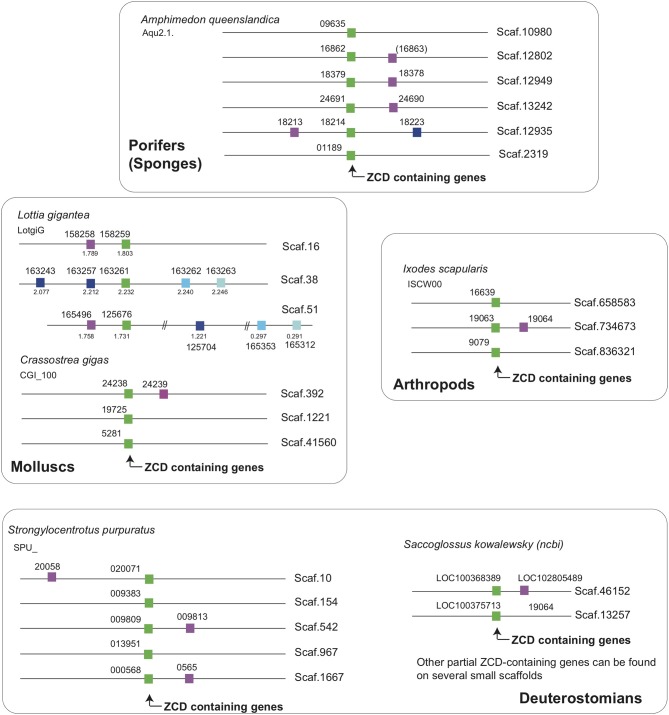
ZCD containing genes are linked to two markers across Metazoa, defining an ancestral synteny. Linkage were determined from Ensembl genome assemblies at Ensembl Metazoa (http://metazoa.ensembl.org/index.html) and at https://www.ncbi.nlm.nih.gov/genome/?term=saccoglossus for *Saccoglossus kowalewsky*, using orthology/paralogy relationships and blast analyses. Homologous genes are indicated by the same color in different species (ZCD containing genes are in green), and gene IDs are associated to each symbol. For Ensembl genes, the prefix for each species is given below the species name (e.g., Aqu2.1 for the sponge *Amphimedon queenslandica*). For the snail *Lottia*, where some paralogs were distant from the reference ZCD containing genes, the coordinates on the scaffolds were added below symbols. Broken lines in a given scaffold indicate a long distance between markers.

Interestingly, two markers located close to ZCD-containing genes from the sponge *Amphimedon queenslandica* had orthologs in the neighborhood of genes encoding ZCD in molluscs, arthropods and echinoderms (Figure 6). These linkages identify an ancestral association of ZCD containing genes with other markers that support a unique common origin.

**Figure 7:**
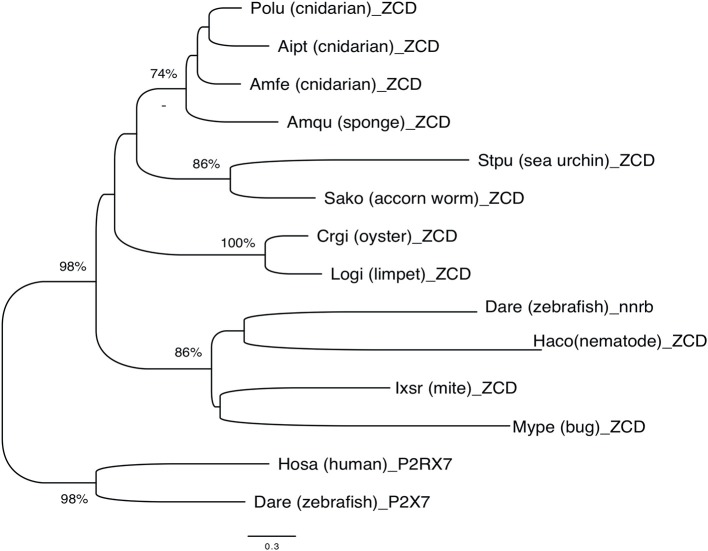
Phylogenetic tree of ZCD motifs from representative Metazoa. The optimal tree (Neighbor-Joining method, 100 bootstrap) is shown. The distances were computed by Mega6 using the JTT matrix-based method and are in the units of the number of aa substitutions per site. Sequences are from Deuterostomes (Hosa_P2X7, human; Rano_P2X7, rat; Aime_P2X7, panda; Dare_P2X7, zebrafish; Stpu_ZCD, sea urchin; Sako_ZCD, accorn worm, XP_002733146), Protostomes [Crgi_ZCD, oyster (Cg_CGI_10024238); Logi_ZCD, limpet (LotgiG163261); Ixsc_ZCD, mite (XP_029850345), Mype_ZCD, a hemiptera insect (XP_022182386); Haco_ZCD, a *Haemonchus* nematode (VDO68347)] and basal groups [Amqu_ZCD, sponge; Polu_ZCD, *Porites lutea* (Cnidaria); Aipt_ZCD, *Aiptasia* sp. (Cnidaria) and Amfe_ZCD, *Amplexidiscus fenestrafer* (Cnidaria)].

Overall, phylogenetic analysis of ZCD containing protein sequences identified groups corresponding to the taxonomic divisions (sponges and cnidarians, mollusks, arthropods). Interestingly, zebrafish nanor-b clustered with arthropods sequences, and not with ZCD from deuterostomians, or with ZCD from vertebrate P2X7 (Figure 7).

### The P2X7 Cytoplasmic Tail: LPS Binding Domain, TNFR Domain, and Others

In human and murine P2X7 sequences, Denlinger et al. (46) reported that the end of exon 13 contains a motif [positions 573–590] similar to the LPS binding domain of the LPS binding protein (LBP) and of the bactericidal/permeability-increasing protein (BPI). They showed that the corresponding peptide bind LPS *in vitro*, and could block LPS-mediated activation of ERK kinases in RAW 264.7 macrophages (46). Thus, these authors proposed that the F, W, and G and the basic conserved residues were critical for the function of the receptor. To study the evolution of this motif, we aligned sequences of human P2X7, LBP, and BPI with fish P2X7, LBP, and BPI, and with other ZCD (Figure 5). While the consensus proposed by Denlinger et al. based on human and murine P2X7, LBP, and BPI is not fully consistent with fish sequences, our alignment indicates that a **WRIR**x(5)**G** consensus is conserved across P2X7 sequences, but not in the other ZCD containing proteins. In rat, this putative LPS binding region overlaps with residues of the high-affinity guanosine nucleotide binding site: R_546_-H_547_, and R_574_-R_578_-K_583_. As across vertebrate P2X7, R_546_ is remarkably conserved across ZCD containing proteins, but the other residues involved in GDP binding in rat are not found in those sequences (Figure S4). The presence of a LPS binding domain in the ballast domain is not obviously supported by recent structural data (47), and direct experiments with proteins from multiple species would have to be done to clarify this point.

In addition to the ZCD canonical cysteine based motif, two other motifs were strikingly conserved across Metazoa: the Y_550_ [position from human P2X7 (41, 42)], and the F/Y_581_P_582_ [positions from human P2X7 (43); Figures 1, 3, and 5]. Interestingly, tyrosine phosphorylation of HSP90 was significantly increased when this protein was associated to the P2X7 mutant Y_550_ F, compared to the wild-type complex (41). In contrast, β arrestin binding sites [T_357_YSS, I_507_TTS, A_540_TNS in human P2X7 (42)] were not conserved: only I_507_TTS was found beyond mammals, but it was not present in nanor-like proteins or in other ZCD outside deuterostomians.

Other motifs were proposed [reviewed in (48)]. They include a potential Src homology 3 (SH3) Domain binding region somewhat similar to the death domain of TNFR, with a PxxP motif located on the 5'side of exon 13 (position 441) (46). These motifs are not conserved in other ZCD containing proteins and their presence is not supported by the recent report of the rat P2X7 structure.

Interestingly, several single nucleotide polymorphisms of the human P2X7 gene have been identified [reviewed in (44)]. Three loss of function polymorphisms have been discovered in the human exon 13: Q460R, E496A, and I568N. We compared these variable sites across human P2X7 haplotypes with the corresponding sites in sequences found in multiple species, to get insights into their level of evolutionary conservation.

Homozygous E496A substitution leads to a significant reduction of P2X7 function in multiple leucocytic cells, with loss of P2X7-dependent non-selective pore formation (49). As seen in Figure 1, E496 is strictly conserved in all vertebrate P2X7 sequences emphasizing the importance of this residue for P2X7 signaling (49). In addition, E496 is also conserved in nanor and frog ZCD (Figure 3) as well as in ZCD across Metazoa (Figure 5). Intriguingly, E496A is a relatively frequent substitution in human P2X7 (45).

I568 is also an important amino-acid because its mutation to N (I568N) inhibits P2X7 plasma membrane expression and normal trafficking (50). I568 forms a di-leucine trafficking/sorting motif (-LL or -IL) (51). This residue is conserved or replaced by another hydrophobic residue, valine, in most P2X7 vertebrate sequences (Figure 1) as well as in ZCD across Metazoa (Figure 5). In human, this substitution is uncommon, being found in 2–3% of the Caucasian population (50). Altogether, this suggests that I568 is highly conserved across species and within the human population.

Finally, the association of the human Q460R polymorphism with bipolar and depressive disorders remains highly controversial as discussed in Stokes et al. (45). Residue Q460 is not well-conserved in vertebrate P2X7 sequences and ZCD domains across Metazoa.

Altogether, our data underscore the conservation of the ZCD across Metazoa sequences similar to P2X7 exon 13, and suggest that it may constitute the primordial pattern of the so-called ballast domain.

## Discussion

Our data indicate that an ancient domain containing a conserved Zn-coordinating cysteine-based motif has been captured by a P2X4-like sequence during vertebrate evolution—possibly after the divergence of agnathans and jawed vertebrates and before the divergence between bony fish and tetrapods. Our data provide an evolutionary perspective about the variation and functional importance of the intracellular C-terminal part typical of the purinergic receptor P2X7.

### Origins of P2X7 ZCD

Our data show that the ZCD motif present in the last exon of P2X7 is an ancient domain already present in the proteome of Sponges and Cnidaria (Sea anemones, jellyfish, and corals). The cysteine based motif coordinating two Zn ions (33) is strikingly conserved not only in all P2X7, but also in all other ZCD sequences we found from sponges to fish across the main groups of Metazoa: sponges, cnidaria, arthropods and nematodes, molluscs, and deuterostomians (echinoderms, acorn worms, and vertebrates). This signature is designed as PTHR36981 in the PANTHER (Protein ANalysis THrough Evolutionary Relationships) protein Classification System, and comprise P2X7, NANOR-like proteins, and unnamed proteins from multiple groups of Metazoa. This Panther ID (http://www.pantherdb.org/panther/family.do?clsAccession=PTHR36981) was not associated to a biological function. Besides P2X7, the only ZCD containing gene for which functional information is available is the zebrafish *nnr* (*nanor*), a zygotic gene expressed at the midblastula transition: the presence of a myristoylation site and Zn-coordinating motifs in NANOR led to the hypothesis of a role in transcription regulation (52). We were unable to detect this domain in fungi, plants, or bacteria. It was not found either in *Monosiga brevicollis*, a choanoflagellate; these protozoans are similar to the choanocytes of sponges, and constitute close relatives of Metazoa. Hence, ZCD seems to be a generic invention of Metazoa like a number of other domains (53).

ZCD are mostly present in relatively short proteins which do not contain other known domains, with the exception of P2X7 and two other proteins from the mite *Ixodes scapularis*. Although sequences of ZCD containing genes seems to be partial in many genomes and should be confirmed in future assemblies, several paralogs were generally found in most species, as in the snail *Lottia*. We could not find well-conserved synteny blocks shared by all ZCD containing genes. P2X7 is located in a relatively stable genomic context across jawed vertebrates. In contrast, nanor-like genes found in many bony fish and in hagfish do not appear to be part of a conserved synteny block. Interestingly, ZCD containing genes were found in association with two closely linked markers across invertebrates, from sponges to mollusks and sea urchin. Overall, our observations indicate that this association is probably the ancestral configuration, which was disrupted by later recombination and duplications. While our observations suggest that ZCD domains have been duplicated multiple times during evolution, they apparently did not expand into large multigenic families. Interestingly, these proteins seem to have been lost in entire groups of animals, such as holometabole insects as well as in smaller taxonomic groups; also, nanor-like genes were apparently absent from many species of bony fish. Besides, they were not seen in basal phyla such as placozoa and ctenophores.

### Are ZCD Conserved GDP-Binding Domains?

The recent report by McCarthy and colleagues demonstrates that P2X7 cytoplasmic ballast domain contains a high-affinity guanosine binding site (33). Our data raise the issue of the conservation of this site in ZCD/ballast-like domains, which we found across Metazoa. The residues interacting with GDP in rat P2X7 (R_546_H_547_R_574_R_578_K_583_) are not all conserved in P2X7 of other species: while R_546_H_547_ R_578_ are generally present except in frog sequences, the two other residues are lost outside mammals. In other ZCD, only R_546_H_547_ and R_578_ were conserved in sea urchin and in the accorn worm *Saccoglossus*, and only R_546and_ R_578_ beyond deuterostomians. Interestingly, molecular models of this region suggests that the two alpha helix in which these key residues are located are overall conserved in all ZCD, as well as the position of the two conserved Arginine. Further work will be required to demonstrate that the ZCD/ballast like domains of fish P2X7, nanor-like proteins, and other ZCD containing proteins from non-vertebrates do indeed bind GDP. This would represent a significant addition to the repertoire of membrane guanine nucleotide binding proteins involved in signal transduction.

### Evolution of the Poly-Palmitoylated Motif

In rat P2X7, at least four cysteines (C362, C363, C374, and C377) and one serine (S360) are palmitoylated in the region 360–377 named C-cys anchor by McCarthy et al. (33). This 18-AA region starts where the second TM region enters the cytoplasm, and anchors the protein to the intracellular face of the plasma membrane. Importantly, the palmitoylated residues within this region are required to maintain a specific property of P2X7, the absence of desensitization after ATP stimulation. This characteristic, which is not observed for other P2X, does not involve the ballast domain. Indeed, when transfected into Xenopus oocytes, a P2X7 lacking the ballast domains displays ATP binding curves, ion selectivity, and lack of desensitization comparable to P2X7 WT (33). In contrast, P2X7-ΔC-cys lacking the C-cys anchor - or P2X7 in which each palmitoylated residues was mutated to alanine–desensitized very quickly after ATP stimulation (33). Interestingly, our comparison of P2X7 sequences across vertebrates show that the C-cys-anchor, encoded by exon 11, is not highly conserved since the number of cysteines is variable and reduced to only one in several fish species. Interestingly, zebrafish or seabream P2X7 receptors transfected in HEK293 cells were unable to desensitize after stimulation by ATP or BzATP, as observed for mammalian P2X7 (39). Furthermore, this lack of desensitization was also found when seabream P2X7 constitutively expressed by a seabream fibroblast line was stimulated (39). These results strongly suggest that the palmitoylation of a unique cysteine can be sufficient to maintain a lack of desensitization of the fish P2X7. Alternatively, palmitoylation of the serine and threonine residues located upstream of the cysteine in fish P2X7 sequences could create a poly-palmitoylation anchor and lead to lack of desensitization. Overall, our data support the idea that palmitoylation and lack of desensitization, which constitute specific features of P2X7, have been added to this receptor independently of the capture of the ancient ballast domain.

## Conclusion

**Figure 8:**
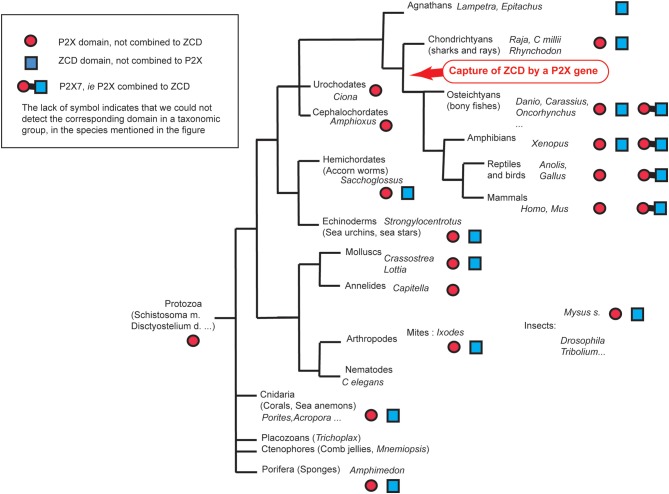
Overview of ZCD containing genes found across Metazoa.

P2X7 is found across vertebrates from bony fish to mammals combining a P2X domain, a putative C-cys anchor and a ballast domain while the other P2X receptors lack the two last features. The conserved genomic co-location of P2X7 and P2X4 genes, with highly similar P2X domain sequences, indicate that they were likely produced by local gene duplication of a unique ancestral gene. Our work suggests that P2X7 originated from the fusion of a P2X4-like gene and a ZCD coding exon during the early evolution of bony fish and tetrapods common ancestors (Figure 8). These domains were connected by a region in which the C-cys anchor critical for the P2X7 properties of desensitization evolved, to produce this unique purinergic receptor critically involved in immunity and inflammation. Our data are important because they demonstrate that the new GDP binding ballast domain identified by McCarthy et al. (33) originates in an ancient family of proteins present across all Metazoa.

## Data Availability Statement

All datasets generated for this study are included in the article/Supplementary Material.

## Author Contributions

AR, OS, SR, JK, and PB conceived the project, designed experiments and approaches, and edited the manuscript. AR, SR, JK, and PB performed primary data analysis. AR, JK, and PB wrote the manuscript.

### Conflict of Interest

The authors declare that the research was conducted in the absence of any commercial or financial relationships that could be construed as a potential conflict of interest.
