# Supplementary material for: Evolutionary Origin of the P2X7 C-ter Region: Capture of an Ancient Ballast Domain by a P2X4-Like Gene in Ancient Jawed Vertebrates
Source: Front Immunol. 2020 Feb 6;11:113. doi: 10.3389/fimmu.2020.00113 (PMC7016195; doi:10.3389/fimmu.2020.00113)
Supplement: Supplementary file 1 [file Image_1.pdf]

Figure S1.

A. Multiple alignment of P2X7 sequences from representative vertebrate species. Exon limits in human P2X7 are boxed and highlighted in red, the TM regions are highlighted in yellow and underlined. **Sequences: Mammals**: Human, Homo sapiens (hosa) ; Mouse, Mus musculus (mumu) ; Rat, Rattus norvegicus (rano). **Reptiles and birds** : Carolina anole, Anolis caroninensis (anca) ; Chicken, Gallus gallus (gaga). **Amphibians** : Clawed frog, Xenopus laevis (xela) ; Xenopus tropicalis (xetr1 : ENSXETG00000001030, xetr2 : ENSXETG00000001030) **Crossopterygians** : Coelacanth, Latimeria chalumnae (lach). **Bony fishes** : Spotted gar, Lepisosteus ocelatus (leoc); Rainbow trout Oncorhynchus mykiss (Onmy ; XP_021461359); Zebrafish, Danio rerio (dare) ; Barramundi perch, Lates calcarifer (laca); Makobe Island cichlid, Pundamilia nyererei, (puny); Sunfish, Mola mola (Momo).

B. **Multiple alignment of human P2X sequences.**

The P2X conserved motif (IPR001429 https://www.ebi.ac.uk/interpro/beta/entry/InterPro/IPR001429/) is underlined, and the exon limits highlighted in green. In the p2X7 sequence, the 2nd transmembrane region is highlighted in yellow.

A.

```
              |  exon1                                  |  exon2
hosa          MPACCSCSDVFQYETNKVTRIQSMNYGTIKWFFHVIIFSYVCFALVSDKLYQRKEPVISS 60
mumu          MPACCSWNDVLQYETNKVTRIQSTNYGTVKWVLHMIVFSYISFALVSDKLYQRKEPVISS 60
rano          ---MLPVRHLCSYNSAKVLHIHSTRLGALKNFFLLAICIYICFALMSDKLYQRKEPLISS 57
anca          MAACSSLKNICEYETNKVVRFQSIFYGSLKWAIHTVVFTYVSVVLIADKRYQKKDNVISS 60
gaga          MVAWGWMKDVFNYESPKLIRFPSVGLVCVKWFIYGVIAVYICYTLIVHKRYQEKEELTSS 60
xela          --MTLTLADCFDYSTKKEVRIQSVPLGILKQCITFGVIVFVCFSLITQKRYQKKDSIISS 58
xetr1         --MAPTFADCFDYSTKKEVRIQSVPLGVLKCCITFGVIVFVCFSLITQKSYQKKDSVISS 58
xetr2         --MAPTFADCFDYSTKKEIRIQSVPLGVLKFLITFGVILFVCFSLITQKSYQKKVSVISS 58
lach          MASVCNWKDCCGYETPRVVHIKSVQLGCLKWTLNGVIFIVIALMLFWNKYYQEYDSIISS 60
leoc          --MPCSLLNFCEYETNKLVRIKSIPIGSLKWSINGLILGFICIMLFWNKEYQEYDQVVSS 58
onmy          --MPCKLLNLCEYETHKLVRIQSARLGSLKWTFNGVILMFICIVMFWNKEYQEHDYVVSS 58
dare          --MPCVLLNLCEYDTQKLVKIKSVKLGSLKWTLNGVILMFICIMMLWNKEYQEYDFVVSS 58
laca          -MARCGLLGLCRYETNKLVRIQSVRLGSLKWILNATILLFICIMMLWNREYQEFDLVVSS 59
puny          -MPCCRLRALCQYETNKLVRIQSVRLGSLKWSLNGAILLFICIMMLWNRKYQEFDLVVSS 59
momo          -MACSWFRSLCQYETNKLVRIQSVRLGSMKWGLNGFILLFICIMMLWNREYQEFDLVVSS 59

                                                   |  exon3
hosa          VHTKVKGIAEVKEEIVENGVKKLVHSVFDTADYTFPLQ-G-NSFFVMTNFLKTEGQEQRL 118
mumu          VHTKVKGIAEVTENVTEGGVTKLGHSIFDTADYTFPLQ-G-NSFFVMTNYVKSEGQVQTL 118
rano          VHTKVKGVAEVTENVTEGGVTKLVHGIFDTADYTLPLQ-G-NSFFVMTNYLKSEGQEQKL 115
anca          VHTKVKGVSQT------------DMRIWDTAEYTIPMQ-GINSFFVVTNIIMTESQTQGV 107
gaga          VRVTLKGVAHV-------------DRIWDAAEYTIPTQ-TRDSFFVMTNIIRTENQIQKT 106
xela          VHTKVKGFADA------------HSRIWDTAEYTVPSP-GGDSFFVITNIVKTEGQMQSN 105
xetr1         VHTKVKGFANA------------YSRIWDTAEYTVPSP-GGDSFFVVTNIVKTENQRQDN 105
xetr2         VHTKVKGIADA------------YSRIWDTAEYTVPSP-GGDSFFVVTNIVKTEDQRQSN 105
lach          VTTKVKGVALTN-------NNELDERIWDVADYIFPAQ-GKNTFFVMTNMIITKNQQQRH 112
leoc          VTAKVKGVAVTN-------ITDVGEILWDAADYSGLSQ-GKNSFFVMTNAIVTNKQRQGK 110
onmy          VTAKVKGVAMTS-------LPDVGDIVWDVVDYSGPSQ-GKNSFFVVTNVIVTKNQRQGK 110
dare          VTTKVKGVAKIT-------LPEVGDVVWDVVDYSGPSQ-GKNSFFVATNAIVTKNQKQGN 110
laca          VTAKVKGVAQTH-------LTGIGDLVWDVVDYSGPSQVGKNSFFVVTNVIVTKNQKQGK 112
puny          VTTKVKGVAQAP-------LHEVGNMVWDEVDYSGPWK-GKNTFFVVTNAIVTQNQKQGK 111
momo          VTTKVKGVAQTH-------LPGIGDTVWDVVDYSGASQT-RNSFFVVTNVIVTKNQRQGK 111

                 |  exon4                 |  exon5                     |
hosa          CPEYPTRRTLCSSDRGCKKGWMDPQSKGIQTGRCVVYEGNQKTCEVSAWCPIEAVEEAPR 178
mumu          CPEYPRRGAQCSSDRRCKKGWMDPQSKGIQTGRCVPYDKTRKTCEVSAWCPTEEEKEAPR 178
rano          CPEYPSRGKQCHSDQGCIKGWMDPQSKGIQTGRCIPYDQKRKTCEIFAWCPAEEGKEAPR 175
anca          CPEFPIAKAICSTDRSCMKGGVDPLSNGIQTGKCVQYNATLKTCEVKAWCPVESMKSAPE 167
gaga          CPEYPTAKAICSSDKSCAKGIVDVHSNGVQTGKCVHYNITHKTCEIKAWCPVQGEERPPV 166
```

```
xela        CSELPSQKTICSRDDICKKGLADPQSNGIQTGRCINFNNTLKTCEVSAWCPVESQ-TTPV 164
xetr1       CSELPSQKTICSRDDVCKKGLADPQSNGIQTGRCINFNSTLKTCEVSAWCPVESQ-TTPV 164
xetr2       CPELPRQKTICSRDDVCKKGLADPQSNGIQTGRCINFNSTLKTCEVSAWCPVESQ-TTPV 164
lach        CPEYPSPLTKCSSQRKCEKGRTALHSNGVQTGRCVNYDAFNKSCEVFAWCPVEAIRAAPV 172
leoc        CAEVPGSGKICYRDQDCTKGYSDQHSHGVQTGSCVKYDTTRKTCQVVAWCPIESKNKPPK 170
onmy        CPEVPPYGKVCWTDKDCVKGFWEQNSHGVQTGACVKFDVKQKTCEVSTWCPIETKKTPPK 170
dare        CAEILPNGKLCRTDKDCEKGFSDQHSHGVQTGACVKLEILKKTCEVTAWCPIENKKN-PR 169
laca        CPEAPRNGRLCRTDKDCEKGAWGQQNHGIQTGSCVKFDVLKKTCEIFAWCPVETKTNPPR 172
puny        CAQVPLKGRFCHTDKDCEKGAWDQQKHGIQTGSCVKFDLSKKTCEVSAWCPIETRSTPPR 171
momo        CPEVPQKGRWCRTDKDCEKGAWDQQSHGIQTGSCVKFDVLKKTCEVSAWCPVESKTNPPR 171

             exon 6                   |  exon7
hosa        PALLNSAENFTVLIKNNIDFPGHNYTTRNILPGLNI----TCTFHKTQNPQCPIFRLGDI 234
mumu        PALLRSAENFTVLIKNNIHFPGHNYTTRNILPTMNG----SCTFHKTWDPQCSIFRLGDI 234
rano        PALLRSAENFTVLIKNNIDFPGHNYTTRNILPGMNI----SCTFHKTWNPQCPIFRLGDI 231
anca        PAILRSAENFTVLIKNNIHFPKFNYTTRNISPEFNT----SCTYNERTASSCPIFRLGDI 223
gaga        PAVLRSSEDFTVFIKNNIHFPTFNYTVQNISPKLNT----SCKFNKVTAPLCPIFRLGDI 222
xela        PAVLESAENFTVLIKNNIHFAAFNFTKKNILPNYNV----SCIYDRVKAPLCPIFRLGDI 220
xetr1       PAVLESAENFTVLIKNNIHFAAFNFTKKNILPKYNV----SCIYDRVKAPLCPIFRLGDI 220
xetr2       PAVLESAENFTVLIKNNIHFAAFNFTKKNILPKYNV----SCIYDRVKAPLCPIFRLGDI 220
lach        PALLRGAEKFSVLIKNNIRFPEFNYTKRNILPHVNETYLKTCTFNRTRDPHCPVFILGNI 232
leoc        PALLESAENFTVLIKNNIRFPAFNYIKRNILPHMNDTYLKSCMFSRRMDPLCPILRLGDI 230
onmy        PALLASAENFTVLIKNNIRFPNFNFIRRNILPEMTDSYLKHCQFNRRTDPWCPIFRLGDI 230
dare        PALLAAAENFTVMIKNNIRFPAFNYIRRNILSEMKDTDFKGCIYHRYKNPYCPIFRLGDI 229
laca        PALLAAAENFTVLIKNNIRFPAFNYFRRNILPEMNDAYLKSCN--RANDSLCPIFRLGDM 230
puny        PALLAAAENFTVLIKNNIRFPAFNYIRRNILPEMNDAYLRNCH--RGNDSLCPIFRLGDI 229
momo        PALLAAAENFTVLIKNNIRFPAFNFIRRNILPEMTDAYLKSCH--RRNDSLCPIFRLGDI 229

                                                     | exon8
hosa        FRETGDNFSDVAI------------------------QGGIMGIEIYWDCNLDRWFHHCR 270
mumu        FQEAGENFTEVAV------------------------QGGIMGIEIYWDCNLDSWSHHCR 270
rano        FQEIGENFTEVAVQLLKLFLRELRQKAEKALDAVPWIRGGIMGIEIYWDCNLDSWSHRCQ 291
anca        LQAIGENFSEVAV------------------------QGGIMGIEINWDCNLDKWRHRCG 259
gaga        LQEAKENFSEMAV------------------------KGGIIAIEIKWDCDLDSWSYYCS 258
xela        LREAGENFSQVAV------------------------LGGVIGIEINWDCDLDPLRYKCE 256
xetr1       LREAGENFSQVAV------------------------LGGVIGIEINWDCDLDSLRYKCE 256
xetr2       LREAGENFSQVAV------------------------LGGVIGIEINWDCDLDSLRYKCE 256
lach        FQEAGENFSEVAI------------------------QGGVMGIQINWDCNLDNWNHKCV 268
leoc        VQEARENFSQMAV------------------------EGGVIGIQINWHCNLDRFFHKCL 266
onmy        VQEARENFSEMAV------------------------EGGVIGIQIKWDCNLDGFLRNCL 266
dare        VAEAKEKFSEMAV------------------------EGGVIGIQINWDCDLNRFFHSCL 265
laca        VRDAGEKFSEMAI------------------------EGGVIGILIKWDCNLDRLMQRCL 266
puny        VREAGERFSEMSV------------------------EGGVIGILIKWDCNLDRLMQRCL 265
momo        VQEAGDKFPEIAI------------------------EGGVISIQIKWDCNLDPLMWRCL 265

                                   |  exon 9                    | exon 10
hosa        PKYSFRRLDDKTTNVSLYPGYNFRYAKYYKEN-NVEKRTLIKVFGIRFDILVFGTGGKFD 329
mumu        PRYSFRRLDDKNTDESFVPGYNFRYAKYYKEN-NVEKRTLIKAFGIRFDILVFGTGGKFD 329
rano        PKYSFRRLDDKYTNESLFPGYNFRYAKYYKEN-GMEKRTLIKAFGVRFDILVFGTGGKFD 350
anca        PKYGFRRLDDKKTSEALYPGYNFRYARYYKQASGREERTLIKAYGIRFDILVFGMAGQFD 319
gaga        PEYSFRRLDDKTR--TQYPGFSIRFARHYKLPDGTEQRTLFKAYGIRFDVLVFGMGGQFK 316
xela        PHYGFRRLDDTVVDESLYPGLNFRFARYYKNAHGTETRTLIKAYGIRFDIQVYGTGGQFN 316
xetr1       PHYSFRRLNNKVVDERLYPGLNFRFARHYKTSDGKDTRTLIKAYGIRFDIQVYGTGGKFS 316
xetr2       PHYSFRRLDDKVVDERLYPGLNFRFARYYKTSDGKETRTLIKAYGIRFDIQVYGMGGKFN 316
lach        PEYTFRRLDNKNANETLSPGYNFRFAQFYKFPNGTEERTLIKAYGIRFDVMVFGTAGRFS 328
leoc        PEYSFRRLDEKESNKTLYPGLNFRFARY-NMNNGIEERMLFKAFGIRFDVMVFGKAGRFS 325
onmy        PKYSFRRLDEKESNRTLSPGLNLRFARY-QTEKGVGERTLFKAFGIRFDVMVFGKAGKFS 325
dare        PKYSFRRLDEKESNRTLYPGLNFRFARY-STVNGVEQRTLFKMYGIRFDVMVFGKAGKFS 324
laca        PRYSFRRLDEKESNRTLYPGINFRYAKY-NTVSGVEERTLYKVFGIRFDVMVFGQAGKFS 325
puny        PRYSFKRLDEKESNKTLYPGLNFRFAKY-NTVNGVEERTLYKAFGIRFDVMVFGQAGKFS 324
momo        PKYSFRRLDEKESNKTLYPGLNFRYAKY-NTVNGVEERTLYKVFGIRFDVMVFGQAGKFS 324

                            |  exon11      <  C-Cyst anchor >
hosa        IIQLVVYIGSTLSYFGLAAVFIDFLIDTYSSNCCRSHIYPWCKCCQPCVVNEYYYRKKCE 389
mumu        IIQLVVYIGSTLSYFGLATVCIDLLINTYSSAFCRSGVYPYCKCCEPCTVNEYYYRKKCE 389
rano        IIQLVVYIGSTLSYFGLATVCIDLIINTYASTCCRSRVYPSCKCCEPCAVNEYYYRKKCE 410
anca        FFELLVYIGSALSYFGLAQLTVDFLITSYSYSCCK-----------YDPVKEYYYKKKCE 368
gaga        LIELFTFIGSTIAYFGLAVTIIEMCFHLYNCSSC----------C-KIQVCENVIRKKYE 365
```

```
xela        LLELALFIGSCLSYFGCASFAIDFIIGRYNSCCCN-----------AKSVLKYYDDRKYE  365
xetr1       LFELAIFIGSCLSYFGCASLAIDFIIGLYKPCCCN-----------AKSVLKYYNDRKYE  365
xetr2       LFELAIFIGSCLSYFGCASLAIDFIIGLYKPCCCN-----------AKSVLKYYDDRKYE  365
lach        FVALILYIGSTLSYYGLTALLIDLLITTYFCPSCP-----------SKEIKDYYLKKIYK  377
leoc        IIQLVIYIGSTLSYYALTTVFIDWFITTYLYPRCC-----------ARATKNNYSEKKYE  374
onmy        IIQLIIYIGSTLSYYALTTLFIDWLIGTS----CF-----------SKEARQNYSEKKVE  370
dare        IIQLIIYIGSTLSYYAITTIFLDWLIGTG----CY-----------SKEAKQNYTERKFE  369
laca        FIQLVIYIGSTLSYYALTSIVLDWLIGTS----CY-----------SAEVGQNYSEKKVE  370
puny        FIQLIIYIGSTLSYYALTTILIDWLIVTS----CY-----------SAEVGQNYSERKVE  369
momo        FIHLIIYIGSTLSYYALTTILIDWLIGTS----CY-----------SAEVGQNYSDRKVE  369



                   |  exon 12                                 |  exon13
hosa        SIVEPKP-------TLKYVSFVDESHIRMVNQQLLGRSLQDVKGQEVPRPAMDFTDLS-R 441
mumu        SIMEPKP-------TLKYVSFVDEPHIRMVDQQLLGKSLQVVKGQEVPRPQMDFSDLS-R 441
rano        PIVEPKP-------TLKYVSFVDEPHIWMVDQQLLGKSLQDVKGQEVPRPQTDFLELS-R 462
anca        SVPGPRW-------TVLYVTYVDEPHIFMIDK-LLKTSLQNIKGKIVHRQTGNFKLGP-K 419
gaga        TVLMPEQ--------VILVSYVDKPHITLIKM-PLRTSLQNAEGSIFEDHPVKSYDP--R 414
xela        TIPGPSVSLAHLKAHLKFVSFVDKEDILMVDQ-KLKGSLQLASGPYIQRERFADTKA--- 421
xetr1       MVPGPA-S------QLKFVSFADEEDILMVDN-NLKDSLQFASGRYIQRERFAYTEA--- 414
xetr2       KVPGPTVS------QLKFVSFVDKEDILMVDI-NSRGSLQFASGQHIQRERFEYTEA--- 415
lach        SVDRPTS-------TLLYVSFVDEQNIFVVKG-PLKVRLQDAKSDIVLEHKVKL-----Q 424
leoc        TVEELNQ-------IPLCISFVDEDQIIVVKR-PLKKSLQETKPSSSQHRKPEHSFGRTR 426
onmy        SVQDRQQ-------CILCVSFVDENHIRVVKR-SHRKSLQQVKPMSVHPFKDDIRH--LS 420
dare        AVQDREE-------CFLCVSFVDEDNLRVVKK-SRKKRLQETKPLSLHQRKNELAS--MK 419
laca        SVQDKQK-------CVLCVSYVDENNIRLLKK-SLKKSLQDVKPMSVQPRKEDTGH--LR 420
puny        SIQDQVK-------CILCVSYIDENNLRLIKR-SQKKSLQVIKPMSVQPRKEDPGH--LR 419
momo        SVQDKEK-------CILCVSYVDVEDIHLVKR-SHKKSLQDIKPMSVQPRKDDTGH--LR 419

                                                                   <-------
hosa        LPLALH--DTPPI-PGQPEE----------------IQLLRKE-ATPRSRDSPVWCQCGS 481
mumu        LSLSLH--DSPLT-PGQSEE----------------IQLLHEE-VAPKSGDSPSWCQCGN 481
rano        LSLSLH--HSPPI-PGQPEE----------------MQLLQIE-AVPRSRDSPDWCQCGN 502
anca        LLLKPP--KEARS-PGAPHE----------------LQPLQNERVASPSRERPPWCSCRE 460
gaga        TC-CSH--KSNEKHGAAQSE----------------LRPLT---QSSSSTNCPKWCCCGR 452
xela        KC--------KDSHKQDENE----------------MRLIKG----RSAMLPPAWCKCNK 453
xetr1       NC--------KDSHKQNESE----------------MRLIKG----SSTTLPPAWCKCNK 446
xetr2       KC--------KDSHKKNQTE----------------MRLIKG----SSTTLPPAWCKCNK 447
lach        EHIALLQPNIEMVCV-------------------------RRTCQSSSEHIIPPWCQCGN 459
leoc        VLVNLLQSPSRNA----------------------DTEIQEMQPPKGRGRSAPAWCQCGR 464
onmy        AMAGVLQKGHSI--------D------------K--------QPQALPRPSCPAWCLCGC 452
dare        TLLSVLQCGQSRSEPVQNGQSGGLIVDENLSSRHNGRQNPDTPLLETIQSSSPTWCQCGS 479
laca        AALSLLQPGVDVDHDAQPLLN------------H--------KPEPKPKVERPSWCKCDC 460
puny        AILCLLQPSDDTNDDVQPSLG------------K--------KTDPNAKPHRPAWCMCDS 459
momo        AVLSLLPPCAGSE----RPLI------------H--------KPSPNPKPHLPAWCKCNC 455

            ---- Ballast domain -----------------------------------------
hosa        CLPSQLPESHRCLEELCCRKKPGACITTSELFRKLVLSRHVLQFLLLYQEPLLALDVD-S 540
mumu        CLPSRLPEQRRALEELCCRRKPGRCITTSKLFHKLVLSRDTLQLLLLYQDPLLVLGEE-A 540
rano        CLPSQLPENRRALEELCCRRKPGQCITTSELFSKIVLSREALQLLLLYQEPLLALEGE-A 561
anca        CRPT-----ERYWEQLCCRRKDGDCIAGSALFDQLVLSRPRLEFILLYADPLLDLKAD-D 514
gaga        CQVA-----QKHHEQLCCRKKEGQCITTTYWFAQLVLSRDTLNKALLYEDPFLDLTGH-S 506
xela        CINT-----THLEEQLCCRLEEGECITDTKMFNSLVLNRESLEYAFQYDNPLSKTP---I 505
xetr1       CIDV-----TQPEEQLCCRLGEGQCITDTKIFNYLVLNKEALEYAFQYDNPLSKTP---E 498
xetr2       CIDV-----TQPEEQLCCRLGQGQCITDTEMFKYLVLNKEALEYAFQYDNPLSKTP---E 499
lach        CHES-----SGFQEMLCCRKNSGPCITTSTMFSQFALSQTTLEFILLYNDPLLDLHAN-R 513
leoc        CQPM-----PTPQEQLCCRRSKGQCITTSPAFPRLVLDRHTLESALLYREPLLELPQA-R 518
onmy        CLPS-----THPQEQLCCRMSRGRCITTSSLFEQLVLSRPLLEAVLLYREPLCELTEGEQ 507
dare        CRPA-----ETLQEQLCCRLKKGRCITSSPIFSSLIVSRSVLENALFFVDPLAELHEE-- 532
laca        CAPS-----SLLQEELCCRQSDGACITSSPLFERLVLHRPLLEAVLLYHDPLSAPADR-G 514
puny        CAPS-----SLPEEELCCRRTEGTCITSSPLFGQLVLRRSLLEAVLLYQDPLSPSVKK-L 513
momo        CAPS-----SVPHEEVCCRQSDGACITSSPLFEQLVLCRPLLEAVLLYRDPLSSPPDR-G 509

            -------------------------------------------------------------
hosa        TNSRLRHCAYRCYATWRFGSQ-DMADFAILPSCCRWRIRKEFPKSEGQYSGFKSPY---- 595
```

```
mumu       TNSRLRHRAYRCYATWRFGSQ-DMADFAILPSCCRWRIRKEFPKTEGQYSGFKYPY---- 595
rano       INSKLRHCAYRSYATWRFVSQ-DMADFAILPSCCRWKIRKEFPKTQGQYSGFKYPY---- 616
anca       LNKRLRHCAYEQYIHWRFGE--ELEERAVLPSCCRWKIRDAFPSEKGEYTGFRSQR---- 568
gaga       SNSQLRRIAYKQYIHWRFGSF-ELEDRAIIPSCCRRLIRSTYPKENGNYTGFNLE----- 560
xela       SKEHLRYYAKQKYVEWRFGCRKYMLNFAVIPNCCKTAIETCNLQTEGP------------ 553
xetr1      S-EDLKCYAKQKYIEWRFRCRRYMLDFAVIPSCCKNAIETCNLQTQHPSGALYLPPTHGM 557
xetr2      S-EDLKCYAKQKYIEWRFGCRRYMLDFAVIPSCCKNAIETCNLQTQHPSGALYLPPTHGM 558
lach       DKDMLRHCAYQQYIHWRFGG-LEKENCAVIPSCCIWKIRETYPSLDGQYTGFRGC----- 567
leoc       CHGHLRHCAYSLYIEWRFGR-IQTGTFAAVPSCSVWRIRDEYPSEDGRYSGLCYENMVII 577
onmy       WTTAMRHCAYNQYITWRFGA-PPRESLPVIPSCSVWRIREEYSSQDGEYTGMRPRRTTSP 566
dare       --SQLRHGAYAQFIRWRFGDSTPRDALPVIPSCCIWRIRAEYPSPDGTYRGLRSFQVITS 590
laca       RTAALRHCAYRQYMSWRFGD-PPVDTQPAIPSCCVWRVREEYPSPDGQYSGFKPTRMVSM 573
puny       QTAALRHCAYREYISWRFGV-PPNDTHPAIPSCCVWRIRREYPSPDGQYSGFRPVKMTSM 572
momo       QTAALRHCAYGQYISWRFGV-TPKDSQPVIPSCCTRRVREEYPSPDGQYSGLRPARLASM 568


           -------->
hosa       -------- 595
mumu       -------- 595
rano       -------- 616
anca       -------- 568
gaga       -------- 560
xela       -------- 553
xetr1      C------- 558
xetr2      C------- 559
lach       -------- 567
leoc       SSNG---- 581
onmy       QSSPNT-- 572
dare       QTEVNR-- 596
laca       QACANGEV 581
puny       QACENGEL 580
momo       QACKNGEL 576
```

B.

```
hP2RX1      ------------MARRFQEELAAFLFEYDTPRMVLVRNKKVGVIFRLIQLVVLVYVIG--46
hP2RX2      MAAAQPKYPAGATARRLARGCWSALWDYETPKVIVVRNRRLGVLYRAVQLLILLYFVW--58
hP2RX3      ------------------MNCISDFFTYETTKSVVVKSWTIGIINRVVQLLIISYFVG--40
hP2RX4      -------------MAGCCAALAAFLFEYDTPRIVLIRSRKVGLMNRAVQLLILAYVIGCY47
hP2RX5      ------------MGQAGCKGLCLSLFDYKTEKYVIAKNKKVGLLYRLLQASILAYLVV--46
hP2RX6      ---MCPQLAGAGSMGSPGATTGWGLLDYKTEKYVMTRNWRVGALQRLLQFGIVVYVVG--55
hP2RX7      -------------MPACC--SCSDVFQYETNKVTRIQSMNYGTIKWFFHVIIFSYVC---42

hP2RX1      --------------WVFLYEKGYQTSSGLI-SSVSVKLKGLAVT-------QLPGLGPQV84
hP2RX2      --------------YVFIVQKSYQESETGPESSIITKVKGITTS------------EHKV92
hP2RX3      --------------WVFLHEKAYQVRDTAIESSVVTKVKGSGL------------YANRV74
hP2RX4      HPHLAEVEMESPRRWVFVWEKGYQETDSVV-SSVTTKVKGVAVT-------NTSKLGFRI99
hP2RX5      --------------WVFLIKKGYQDVDTSLQSAVITKVKGVAFT-------NTSDLGQRI85
hP2RX6      --------------WALLAKKGYQERDLEPQFSIITKLKGVSVT-------QIKELGNRL94
hP2RX7      --------------FALVSDKLYQRKEPVI-SSVHTKVKGIAEVKEEIVENGVKKLVHSV87

hP2RX1      WDVADYVFPAQGDNSFVVMTNFIVTPKQTQGYCAEHPEG--GICKEDSGCTPGKAKRKAQ142
hP2RX2      WDVEEYVKPPEGGSVFSIITRVEATHSQTQGTCPESIRVHNATCLSDADCVAGELDMLGN152
hP2RX3      MDVSDYVTPPQGTSVFVIITKMIVTENQMQGFCPESEEK--YRCVSDSQCGPER--LPGG130
hP2RX4      WDVADYVIPAQEENSLFVMTNVILTMNQTQGLCPEIPDAT-TVCKSDASCTAGSAGTHSN158
hP2RX5      WDVADYVIPAQGENVFFVVTNLIVTPNQRQNVCAENEGIPDGACSKDSDCHAGEAVTAGN145
hP2RX6      WDVADFVKPPQGENVFFLVTNFLVTPAQVQGRCPEHPSVPLANCWVDEDCPEGEGGTHSH154
hP2RX7      FDTADYTFPLQG-NSFFVMTNFLKTEGQEQRLCPEYPTRR-TLCSSDRGCKKGWMDPQSK145

hP2RX1      GIRTGKCVAF-NDTVKTCEIFGWCPVEVDDDIPRPALLREAENFTLFIKNSISFPRFKVN201
hP2RX2      GLRTGRCVPYYQGPSKTCEVFGWCPVEDGASVS-QFLGTMAPNFTILIKNSIHYPKFHFS211
hP2RX3      GILTGRCVNY-SSVLRTCEIQGWCPTEVDTV--ETPIMMEAENFTIFIKNSIRFPLFNFE187
hP2RX4      GVSTGRCVAF-NGSVKTCEVAAWCPVEDDTHVPQPAFLKAAENFTLLVKNNIWYPKFNFS217
hP2RX5      GVKTGRCLRRENLARGTCEIFAWCPLETS-SRPEEPFLKEAEDFTIFIKNHIRFPKFNFS204
hP2RX6      GVKTGQCVVF-NGTHRTCEIWSWCPVESG-VVPSRPLLAQAQNFTLFIKNTVTFSKFNFS212
```

```
hP2RX7      GIQTGRCVVY-EGNQKTCEVSAWCPIEAVEEAPRPALLNSAENFTVLIKNNIDFPGHNYT204

hP2RX1      RRNLVEEVNAAHMKTCLFHKTLHPLCPVFQLGYVVQESGQNFSTLAEKGGVVGITIDWHC261
hP2RX2      KGNIADR-TDGYLKRCTFHEASDLYCPIFKLGFIVEKAGESFTELAHKGGVIGVIINWDC270
hP2RX3      KGNLLPNLTARDMKTCRFHPDKDPFCPILRVGDVVKFAGQDFAKLARTGGVLGIKIGWVC247
hP2RX4      KRNILPNITTTYLKSCIYDAKTDPFCPIFRLGKIVENAGHSFQDMAVEGGIMGIQVNWDC277
hP2RX5      N-NVMDVKDRSFLKSCHFGPK-NHYCPIFRLGSVIRWAGSDFQDIALEGGVIGINIEWNC262
hP2RX6      KSNALETWDPTYFKHCRYEPQFSPYCPVFRIGDLVAKAGGTFEDLALLGGSVGIRVHWDC272
hP2RX7      TRNILPGLNI----TCTFHKTQNPQCPIFRLGDIFRETGDNFSDVAIQGGIMGIEIYWDC260

hP2RX1      DLDWHVRHCRPIYEFHGLY---EEKNLSPGFNFRFARHFVEN-GTNYRHLFKVFGIRFDI317
hP2RX2      DLDLPASECNPKYSFRRLDPKHV--PASSGYNFRFAKYYKIN-GTTTRTLIKAYGIRIDV327
hP2RX3      DLDKAWDQCIPKYSFTRLDSVSEKSSVSPGYNFRFAKYYKMENGSEYRTLLKAFGIRFDV307
hP2RX4      NLDRAASLCLPRYSFRRLDTRDVEHNVSPGYNFRFAKYYRDLAGNEQRTLIKAYGIRFDI337
hP2RX5      DLDKAASECHPHYSFSRLDNKL-SKSVSSGYNFRFARYYRDAAGVEFRTLMKAYGIRFDV321
hP2RX6      DLDTGDSGCWPHYSFQLQE---------KSYNFRTATHWWEQPGVEARTLLKLYGIRFDI323
hP2RX7      NLDRWFHHCRPKYSFRRLDDKTTNVSLYPGYNFRYAKYYKEN-NVEKRTLIKVFGIRFDI319

hP2RX1      LVDGKAGKFDIIPTMTTIGSGIGIFGV--------------------------ATVLCDL
hP2RX2      IVHGQAGKFSLIPTIINLATALTSVGVVRNPLWGPSGCGGSTRPLHTGLCWPQGSFLCDW
hP2RX3      LVYGNAGKFNIIPTIISSVAAFTSVGV--------------------------GTVLCDI
hP2RX4      IVFGKAGKFDIIPTMINIGSGLALLGM--------------------------ATVLCDI
hP2RX5      MVNGK------------------------------------------------GAFFCDL
hP2RX6      LVTGQAGKFGLIPTAVTLGTGAAWLGV--------------------------VTFFCDL
hP2RX7      LVFGTGGKFDIIQLVVYIGSTLSYFGL--------------------------AAVFIDF
             <             transmembrane2 region

hP2RX1      LLLHILP------------------KRHYYKQKKFKYAEDMGPGAAERDLA-ATSSTLGL
hP2RX2      ILLTFMN------------------KNKVYSHKKFDKVCTPSHPSGSWPVT--LA-----
hP2RX3      ILLNFLK------------------GADQYKAKKFEEVNETTLKIAALTNP-VYPSDQTT
hP2RX4      IVLYCMK------------------KRLYYREKKYKYVEDYEQGLASELDQ---------
hP2RX5      VLIYLIK------------------KREFYRDKKYEEVRGLEDSSQEAEDE---ASGLGL
hP2RX6      LLLYVDR------------------EAHFYWRTKYEEAKAPKATANSVWRELALASQARL
hP2RX7      LIDTYSSNCCRSHIYPWCKCCQPCVVNEYYYRKKCESIVEPKPTLKYVSF--VDESHIRM
             &gt;

hP2RX1      QENMRTS-----------------------------------------------------
hP2RX2      RVLGQAPPEPGHRSEDQHPSPPSGQEGQQGAECGPAFPPLRPCPISAPSEQMVDTPASEP
hP2RX3      AEKQSTDSGAFSIGH---------------------------------------------
hP2RX4      ------------------------------------------------------------
hP2RX5      SEQLTSGPGLLGMPEQQELQEPPEAKRGSSSQKGNGSVCPQLLEPHRLRTPSASPLHQE
hP2RX6      AECLRRSSAPAPTATAAGSQTQTPGWPCPSSDTHLPTHSGSL-----------------
hP2RX7      VNQQLLGRSLQDVKGQEVPRPAMDFTDLSRLPLALHDTPPIPGQPEEIQLLRKEATPRS

hP2RX1      ------------------------------------------------------------
hP2RX2      PAQASTPTDPKGLAQL--------------------------------------------
hP2RX3      ------------------------------------------------------------
hP2RX4      ------------------------------------------------------------
hP2RX5      ------------------------------------------------------------
hP2RX6      ------------------------------------------------------------
hP2RX7      RDSPVWCQCGSCLPSQLPESHRCLEELCCRKKPGACITTSELFRKLVLSRHVLQFLLLYQ

hP2RX7      EPLLALDVDSTNSRLRHCAYRCYATWRFGSQDMADFAILPSCCRWRIRKEFPKSEGQYSG

hP2RX7      FKSPY   595
```
