# Supplementary material for: Evolutionary Origin of the P2X7 C-ter Region: Capture of an Ancient Ballast Domain by a P2X4-Like Gene in Ancient Jawed Vertebrates
Source: Front Immunol. 2020 Feb 6;11:113. doi: 10.3389/fimmu.2020.00113 (PMC7016195; doi:10.3389/fimmu.2020.00113)
Supplement: Supplementary file 2 [file Image_2.pdf]

Figure S2. Multiple alignment of P2X7 C Cys anchor (Exon 11) from representative species of mammals and reptiles.

Key residues of the C Cyst anchor are highlighted in yellow, an additonal cystein is highlighted in grey.

Species : **Mammals**: Human, *Homo sapiens* (hosa) ; Mouse, *Mus musculus* (mumu) ; Rat, *Rattus norvegicus* (rano) ; Rabbit, *Oryctolagus cuniculus* (Orcu) ; Bison, *Bison bison (*Bibi*)* ; Cow, *Bos taurus* (Bota) ; Dolphin, *Tursiops truncatus,* (Tutr) ; Sperm whale, *Physter macrocephalus* (Phma) *;* Microbat, *Myotis lucifugus (Mylu*) ; Megabat, *Pteropus vampyrus (Ptva*) ; American mink, *Neovison vison (Nevi*) ; Cat, *Felix catus*, (Feca); Panda, *Ailuropoda melanoleuca* (Aime) ; Elephant *Loxodonta africana* (Loaf) ; Armadillo *Dasypus novemcinctus (Dano*) ; Common wombat, *Vombatus ursinus (Vour*) ; Wallaby *Notamacropus eugenii (Noeu*) ; Opossum, *Monodelphis domestica (Modo*)

Reptiles and birds : Carolina anole, *Anolis carolinensis* (Anca) ; Chicken, *Gallus gallus* (Gaga); Chinese softshell turtle, *Pelodiscus sinensis*, (Pesi) ; Abingdon island giant tortoise, *Chelonoidis abingdonii (chab*).

**Eutherians**	Hosa	AAVFIDFLIDTYSSNCCRSHIYPWCKCCQPCVVNEYYYRKKCESIVEPK
	Mumu	ATVCIDLLINTYSSAFCRSGVYPYCKCCEPCTVNEYYYRKKCESIMEPKP
	Rano	ATVCIDLIINTYASTCCRSRVYPSCKCCEPCAVNEYYYRKKCEPIVEPKP
	Orcu	ATVCIDFLINTYSSNCCRSHIYPRCTCCEPCAANEYYHRKKYESLVEPRR
	Bibi	ATVFIDMLINTYSSKYCRSHVYPWCKCCQPCAVNEYYYKKKYESIVEPTR
	Bota	ATVFIDMLINTYSSKYCRSHVYPWCKCCQPCAVNEYYYKKKYESIVEPTR
	Tutr	ATVFIDFLINTYSNKCCRSHIYPCCKCCEPCKVNEYYYRKKCESIVEPKQ
	Phma	ATVFIDFLINTYSYKCCRSRIYPCCKCCEPCAVNEYYYRKKCESIAEPKQ
	Mylu	ATVFIDFLINTCSNTSCQSYCKCCECCAVNEYYYKKKCESIVEPKS
	Ptva	ATMFIDSLIDTYSSKCCRSRIYPYCKCCECCAVNEYYYRKKCESIVEPKP
	Nevi	VTVIIDLLICTYSSTCCRSHIYPSFKCCKYCAVNEYYYRKKGEPIVEPKR
	Feca	ATLFIDFLINTYSSKCCRSHIYPCFKCCECCAVNEYYYRKKCETIVEPKAT
	Aime	ATVFIDILINTYSSKCCRSRIYPCFKCCEYCAVNEYYYRKQSEPIAEPKP
	Loaf	ATVFIDFLINTYSTECCGK---CVHPYCKRCRVNEYYYKKKCETIVEPKA
	Dano	ATVFIDFLINTYSSGWCQSYVYPCCKCCQPCAVNEYYYRKKCESIVEPKP
	Vour	ATVFIDFLIDTYSSTCCRSRVYRCCPVCEPCGVNEYYYRKKCETIEEPKP
**Marsupials**	Noeu	ATVFIDFLIDTYSSTCCRSSVYHCCPVCEPCGVNEYYYRKKCETIEEPKR
	Modo	ATVFIDFLIDTYSSTCCRTHVYPCCKACEPCGVNEYYYRKKCETIEEPKP
	Gaga	AVTIIEMCFHLYNCSSCC------------KIQVCENVIRKKYETVLMPEQ-
	Anca	AQLTVDFLITSYSYSCC-----------KYDPVKEYYYKKKCESVPGPRW
**Reptiles and birds**	Pesi	AQFLIDFLITSYTFPCC------------KASIKKYYFRKKCESAPGPKWT
	Chab	AQFVIDFLITSYTYPCC------------KASIKEYYFRKKCESALGPKWV
