# Supplementary material for: Evolutionary Origin of the P2X7 C-ter Region: Capture of an Ancient Ballast Domain by a P2X4-Like Gene in Ancient Jawed Vertebrates
Source: Front Immunol. 2020 Feb 6;11:113. doi: 10.3389/fimmu.2020.00113 (PMC7016195; doi:10.3389/fimmu.2020.00113)
Supplement: Supplementary file 3 [file Image_3.pdf]

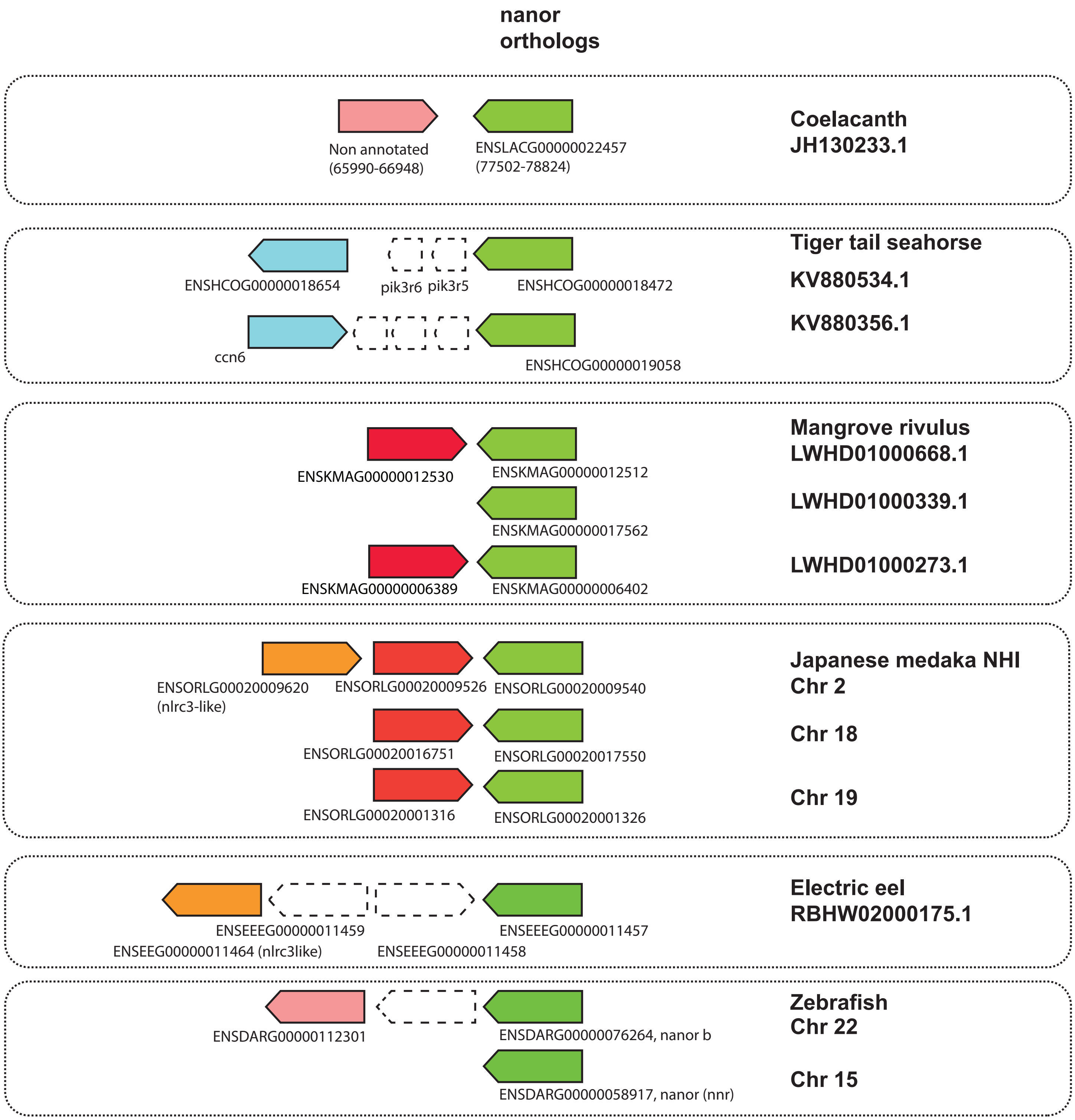

Figure S3. **Nanor-like genes are not included in large conserved synteny blocks**. Phylogenetic relationships between genes located in the neighborhood of zebrafish nnr and nanorb were studied using genomicus and direct blast analysis. Well supported (co)orthology between flanking markers is denoted by red, orange and light blue. Genes represented in pink were significantly similar to medaka ENSORLG00020009526 or to rivulus ENSKMAG00000012530 (tblastn e values < 1e-10) but did not stand in Ensembl as their true orthologs .
