# Supplementary material for: Evolutionary Origin of the P2X7 C-ter Region: Capture of an Ancient Ballast Domain by a P2X4-Like Gene in Ancient Jawed Vertebrates
Source: Front Immunol. 2020 Feb 6;11:113. doi: 10.3389/fimmu.2020.00113 (PMC7016195; doi:10.3389/fimmu.2020.00113)
Supplement: Supplementary file 4 [file Image_4.pdf]

Figure S4. Variable degree of conservation of P2X7 putative LPS and GDP binding motifs in other ZCD containing proteins.

Residues consistent with consensus LBP/BPI from ref Denlinger et al J Immunol 2001; 167:1871-1876 are in bold and blue. Residues not consistent with the consensus are highlighted in grey. Sequences of ZCD containing proteins are as in Figure 5.

```
                                                                       LPS binding motif
Hosa_LPBmotif                                                         GRWKVRKSFFKLQGSFDV
Hosa_BPImotif                                                         GKWKAQKRFLKMSGNKDL
Consensus Denlinger                                                   x+W+hRKxFhKxpGpaph
Dare LBP                                                              GRWRTKFGIITDSGSFDV-

Tetraodon BPI-like                                                    GNWRVKYRWVKNSGSFD

Hosa_P2RX7               NSRLRHCAYRCYATWRFG--SQDMADFAILPSCCRWRIRKEFPKSEGQYSGFKSPY----
Aime_P2RX7               -SRLRHCAYRCYTTWRFG--SPDLADFAILPSCCRWRIRREFPKSEGQYTGFQSPY
Darer_P2X7               -SQLRHGAYAQFIRWRFGD-STPRDALPVIPSCCIWRIRAEYPSPDGTYRGLRSFQVITSQTEVNR
Dare_nanor                          NFIFLCWS-DVGQQTRAVIPSCVAGRMRQKLPEGNENYNGLFPRPL----------
Dare_nanorb                         NIVWWCWG-YLGREIRVVIPSCAVTRIRQEFPDENENYTGYLPRPM----------
Elel_nanor                          KCIICWNREFLGRLSRIPIPSFVVNHIGQTFPNEHGLP--------------
Krma_nanor                          QFVSWCWS-FLGEHIRVVLPSCVVSKIRAFFPPPGLEENFVFTGFLD----------
Stpu_ZCD                 NRAMRHAAYRCFVFWQHG--RLGQGNRRVIPSCCVSEIRGMYPSATVVLRK---------
Sako_ZCD                 NACFRHYAYRQFIYWQYG--RLGKGNRRVVPSCCVWAVRRRFPSPNNVYVGFKEGAIQD-
Amqu_ZCD                 PESFRKAAYRQYILWKYK--KLGKGNRRVCPSCVVNCIREWYPSSTGRYMGFRSE-----
Polu_ZCD                 TQSYRKAAYRQYTLWKFG--KLGRGNRKILPSCVVIVIRRAYPAPDGNYMGFRRS-----
Aipt_ZCD1                TSSFRKAAYRQFILWEHG--YLGKGNRRIIPSCDVKKVRAKYPAPDNVYMGFMAE-----
Aipt_ZCD2                MEGFRKAGYRQFALWRYG--KLGRGNRRILPACVVKRIRAQYPAPDGRYMGYRAN-----
Aipt_ZCD3                MNGYRKTAYRTYILWKYG--KLGRGNRKVVPACVVRAIRRAYPSEDNVYMGFRQS-----
Amfe_ZCD                 VNSYRKAAYRQYILWKYG--KLGKGNRRVCPSCVVRLVRQTYPSPDGEYMGFRRH-----
Crgi_ZCD                 QSTFRNQAYRNFVLWQHS--TLGAGRRVPVPSCVCVAIRRRFPQANGQYMGYHSANSDSE
Logi_ZCD                 NKTFRNQAYRNFILWQHG--KMGSGKRKPLPACTYVAIRATFPSN--EYSGYHSAFSDSE
Ixsc_ZCD                 HERHRYTAYQQFTWWVDH--KPRHGNRVPLPSCAVERIRREFPSTNGSYVDQKK------
Mype_ZCD                 NSSWRFICYTQYTHWINSWSSFGKGVRVVIPACVVKTIREKYPEVNGVYVGFKNGSKLPN
Haco_ZCD                 NRCFRYCAYRSFVIWCYG—RLGILRGFELPACVRGAIMKEFPSDSGSY
```

a:aromatic, h :hydrophobic
p:polar, x :random, -:acidic
and + :basic
